# VO_2_-Based Spacecraft Smart Radiator with High Emissivity Tunability and Protective Layer

**DOI:** 10.3390/nano14161348

**Published:** 2024-08-15

**Authors:** Qingjie Xu, Haining Ji, Yang Ren, Yangyong Ou, Bin Liu, Yi Wang, Yongxing Chen, Peng Long, Cong Deng, Jingting Wang

**Affiliations:** School of Physics and Optoelectronics, Xiangtan University, Xiangtan 411105, China202121521308@smail.xtu.edu.cn (Y.C.);

**Keywords:** VO_2_, smart radiator device, Fabry–Perot resonance, emissivity tunability, protective layer

## Abstract

In the extreme space environment, spacecraft endure dramatic temperature variations that can impair their functionality. A VO_2_-based smart radiator device (SRD) offers an effective solution by adaptively adjusting its radiative properties. However, current research on VO_2_-based thermochromic films mainly focuses on optimizing the emissivity tunability (Δ*ε*) of single-cycle sandwich structures. Although multi-cycle structures have shown increased Δ*ε* compared to single-cycle sandwich structures, there have been few systematic studies to find the optimal cycle structure. This paper theoretically discusses the influence of material properties and cyclic structure on SRD performance using Finite-Difference Time-Domain (FDTD) software, which is a rigorous and powerful tool for modeling nano-scale optical devices. An optimal structural model with maximum emissivity tunability is proposed. The BaF_2_ obtained through optimization is used as the dielectric material to further optimize the cyclic resonator. The results indicate that the tunability of emissivity can reach as high as 0.7917 when the BaF_2_/VO_2_ structure is arranged in three periods. Furthermore, to ensure a longer lifespan for SRD under harsh space conditions, the effects of HfO_2_ and TiO_2_ protective layers on the optical performance of composite films are investigated. The results show that when TiO_2_ is used as the protective layer with a thickness of 0.1 µm, the maximum emissivity tunability reaches 0.7932. Finally, electric field analysis is conducted to prove that the physical mechanism of the smart radiator device is the combination of stacked Fabry–Perot resonance and multiple solar reflections. This work not only validates the effectiveness of the proposed structure in enhancing spacecraft thermal control performance but also provides theoretical guidance for the design and optimization of SRDs for space applications.

## 1. Introduction

When spacecraft operate in space, they pass through sunlit and shadow zones, leading to temperature variations of several hundred degrees Celsius, with extremes from −150 °C to 150 °C [1,2]. To ensure the normal operation of spacecraft, all payloads and equipment must be maintained within a relatively constant temperature range; for instance, the operating temperature of a satellite must be kept between 0 °C and 40 °C, making an effective thermal control system crucial for proper functioning [3,4]. In space, where thermal conduction and convection do not exist, thermal radiation becomes the only way for spacecraft to dissipate heat into outer space [5,6]. The thermal control system of spacecraft reduces temperature variations by adjusting the emissivity of its surface coatings. However, traditional spacecraft thermal control coatings lack the ability to adjust their radiative properties, making it difficult for the spacecraft to adapt to fluctuations in thermal loads and environmental conditions. When the temperature of a spacecraft is high, the emissivity of the coating will increase to enhance surface radiative cooling, effectively lowering the temperature. On the contrary, when the temperature is low, the emissivity of the coating will decrease to minimize radiative heat loss and maintain a stable temperature. This dynamic regulation of radiative properties is achieved through the smart radiator device (SRD) [7].

The performance of an SRD is mainly determined by the difference between the maximum and minimum values of infrared emissivity (*ε*), denoted as Δ*ε* [8,9]. The larger the Δ*ε*, the higher the efficiency of an SRD. VO_2_ is a well-known thermal material with thermally induced infrared emissivity that undergoes a phase transition between dielectric and metallic states near its phase transition temperature (T_C_ = 68 °C), resulting in significant changes in its infrared optical properties [10,11,12,13,14]. It is worth noting that VO_2_ exhibits low emissivity at high temperatures and high emissivity at low temperatures before and after phase transition, which is opposite to the requirements of spacecraft [15,16]. Therefore, VO_2_ is typically combined with other materials to form Fabry–Perot (FP) resonant cavities to meet the diverse temperature requirements of spacecraft [17,18,19].

The commonly used VO_2_-based SRD consists of a sandwich structure, with a highly reflective metal at the bottom, a dielectric resonant cavity in the middle, and an active VO_2_ thin film at the top [8,17,18,19]. The main factors affecting the SRD performance of this sandwich structure are the material’s properties and thickness [9,20,21,22,23]. Previous studies have often focused on analyzing these two factors, with relatively less research on multi-phase layer structures. Most of the existing literature has limited research on the number of cycles, leaving considerable potential for improvement in achieving broad emissivity tuning [18,24].

Furthermore, considering that spacecraft operate in space, the performance and lifespan of such multilayer structures are susceptible to the external space environment. Studies have demonstrated that atomic oxygen can adversely affect the optical properties of VO_2_ coatings, as evidenced by a series of space qualification tests revealing vacuum-accelerated aging [15,25]. In addition to optimizing the material structure to enhance SRD performance, it is also crucial to extend its operational lifespan. Therefore, adding a protective layer without compromising its optical performance to improve its serviceability is urgently needed.

**Figure 1:**
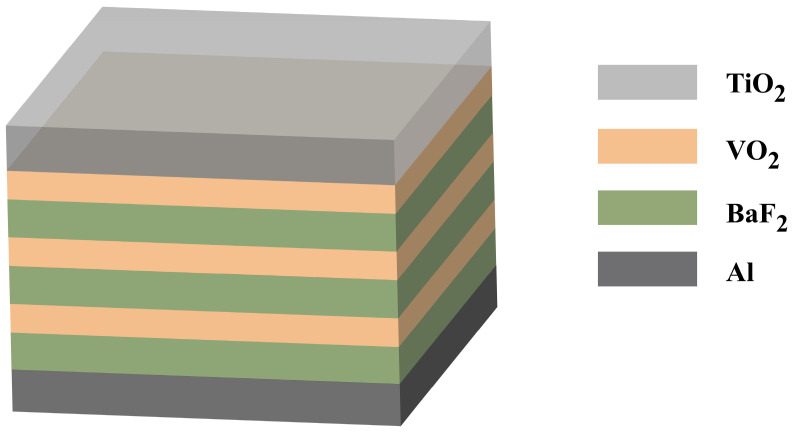
Optimal SRD structure with maximum emissivity tunability.

In this paper, a VO_2_-based spacecraft smart radiator with high emissivity tunability and a protective layer is designed, and its infrared emissivity tunability is systematically optimized in terms of both material properties and multi-periodic structure. Firstly, BaF_2_ was identified as the optimal dielectric layer material. Subsequently, stacking VO_2_ and BaF_2_ layers resulted in a maximum Δ*ε* of 0.7917 when three BaF_2_ layers were alternately scaped with three thin VO_2_ layers on an Al substrate. Furthermore, the impact of protective layer materials (HfO_2_ or TiO_2_) and their thicknesses on the spacecraft’s thermal control performance was investigated. For TiO_2_ as a protective layer, the tunability of infrared emissivity slightly increased before decreasing with thickness, showing an overall minimal change. In contrast, HfO_2_ as a protective layer leads to a decrease in infrared emissivity with increasing thickness, significantly impacting the original structure’s performance. Consequently, the optimal structure proposed in this paper is illustrated in Figure 1. The proposed structure achieves the combination of stacked Fabry–Perot resonance and multiple solar reflections by stacking VO_2_ and BaF_2_ on an Al substrate, maximizing the tunability of emissivity. The protective layer prevents microstructural changes in multilayer composite films caused by atomic oxygen exposure, which helps to extend the lifespan of the thermal control system. Furthermore, the causes of enhanced emissivity tunability are analyzed by the distribution of the electric field. This structure exhibits high emissivity tunability and a long operational lifespan, demonstrating great potential for spacecraft thermal control applications.

## 2. Principle and Simulation

### 2.1. Structure Design

**Figure 2:**
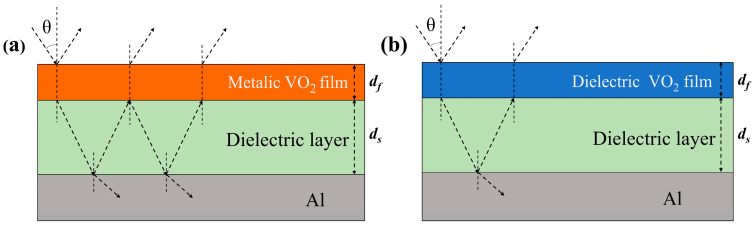
The single-cycle sandwich structure and the light propagation when VO_2_ is in the metallic state (**a**) and dielectric state (**b**), where *d_f_* and *d_s_* represent the thicknesses of the VO_2_ and spacer, respectively.

The basic structure of an SRD is a Fabry–Perot resonator operating at a thermal equilibrium temperature T, which utilizes wave interference to provide absorption enhancement in the mid-infrared band. Figure 2 illustrates the effect of variable emissivity of VO_2_ at different temperatures. At temperatures above 341 K, the metal phase of VO_2_ enhances thermal radiation and generates strong resonance effects within the Fabry–Perot cavity (Figure 2a). The resonator exhibits high broadband emissivity, which contributes to radiative cooling. At temperatures below 341 K, VO_2_ is in its dielectric phase, resulting in no significant Fabry–Perot enhancement (Figure 2b) [26]. Within the considered wavelength range, both the dielectric VO_2_ and the spacer layers appear transparent, leading to high reflectivity of the structure primarily due to the presence of the Al substrate in the low-temperature environment. Therefore, through the analysis of Figure 2a,b, it can be concluded that the single-cycle sandwich structure has low emissivity at low temperatures and high emissivity at high temperatures, which meets the requirements of spacecraft [15,16].

### 2.2. Optical Simulation

To numerically study the proposed structure, we utilize Finite-Difference Time-Domain (FDTD) software (Lumerical FDTD solutions, version 2023 R1) for simulation, aiming to calculate the emissivity and electric field intensity [27]. The FDTD method is a 3D full-wave electromagnetic solver commonly used for modeling nanophotonic devices and processes. In the simulation, the film shapes are set as rectangular, with a length and width of 400 nm. The length and width of the simulation area of FDTD are also set to 400 nm, with a minimum grid of 0.02 and an accuracy of 4. Plane waves are used as the simulated incident light source, incident from top to bottom, with a simulation wavelength range from 2.5 to 25 µm. A perfectly matched layer (PML) is applied in the z-direction to simulate infinite space while using periodic boundary conditions in the x and y directions. Reflectivity is measured by a reflection monitor placed behind the light source, and transmittance is measured by a transmittance monitor placed below the films.

### 2.3. Calculation of the Optical Properties

According to Kirchhoff’s law, the directional emissivity of any object is equal to its absorption under the condition of thermal equilibrium, which can be described as follows [28,29,30,31,32]:

Ɛ = A(1)

where A and Ɛ are absorptivity and emissivity at different temperatures. Assuming that R is the reflectivity, and T is the transmittance, the following relationship is satisfied:

R + A + T = 1(2)

Therefore, the emissivity can be calculated by the following:

Ɛ = A = 1 − R − T(3)

By utilizing the FDTD software to obtain the numerical values of R and T, Ɛ can thus be calculated through Equation (3).

## 3. Results and Discussion

### 3.1. Dielectric Layer Material Optimization

The type and thickness of materials significantly affect the performance of the SRD. Initially, we studied three types of single-cycle sandwich structures, each based on different dielectric layer materials (Al_2_O_3_, ZnS, and BaF_2_). We discussed how the dielectric layer material and the thickness of VO_2_ impact the total emissivity of each structure, and ultimately determined their infrared emissivity tunability Δ*ε*.

**Figure 3:**
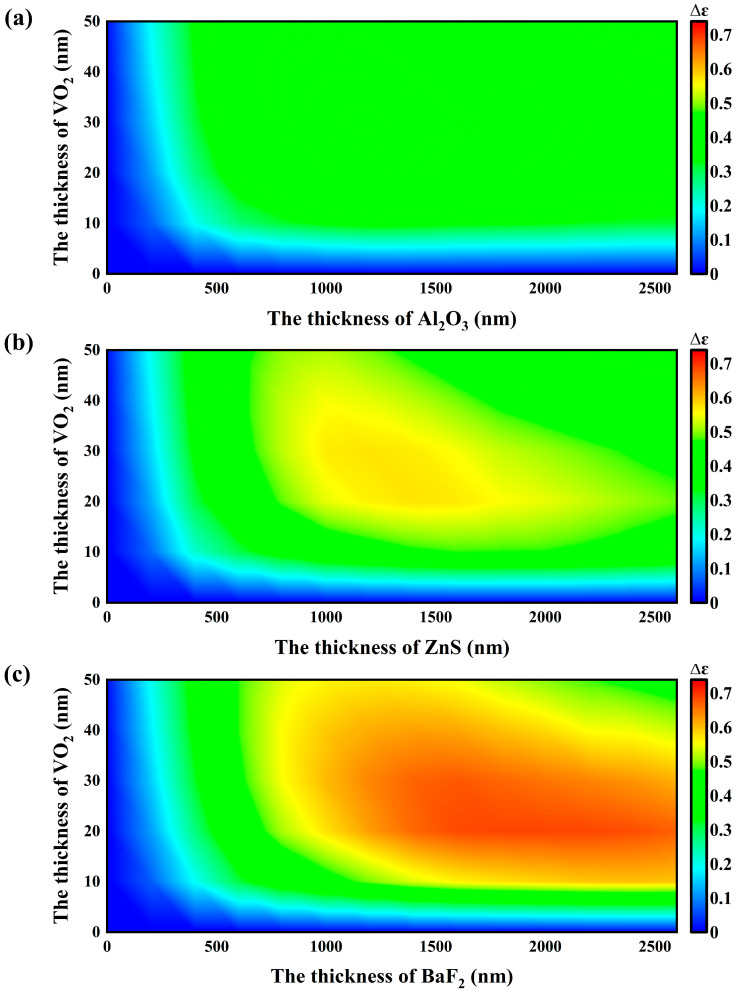
Emission tunability (Δ*ε*) as functions of the thicknesses of VO_2_ and three dielectric layer materials, (**a**) Al_2_O_3_, (**b**) ZnS, and (**c**) BaF_2_.

As shown in Figure 3, the maximum emissivity tunability of Al_2_O_3_, ZnS, and BaF_2_ is 0.4620, 0.5730, and 0.6876, respectively. The structure using BaF_2_ as the dielectric layer achieves the highest tunability of infrared emissivity. At this configuration, the thicknesses of BaF_2_ and VO_2_ are 2000 nm and 20 nm, respectively.

### 3.2. Resonant Cavity Period Optimization

**Figure 4:**
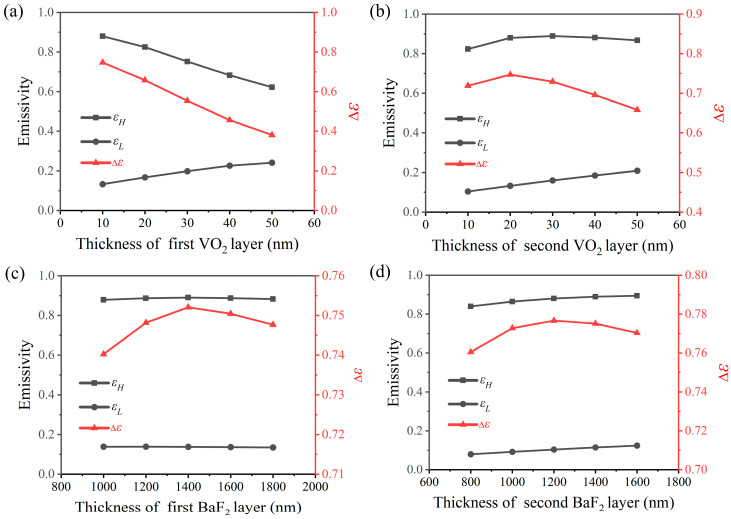
The IR emittance of high/low temperatures and the emissivity tunability (Δ*ε*) of the VO_2_-based two-period structure varies with the thickness of the first VO_2_ layer (**a**), the second VO_2_ layer (**b**), the first BaF_2_ layer (**c**) and the second BaF_2_ layer (**d**).

The single-cycle sandwich structure of the SRD optimized in the previous section is Al/BaF_2_/VO_2_. To further improve the modulation effect, multiple Fabry–Perot resonances are generated by alternately stacked multiple layers of BaF_2_/VO_2_, optimizing the multi-period structure and enhancing the tunability of emissivity. Figure 4 shows the emissivity tunability of a two-period structure based on the stacked FP multilayer film of VO_2_/BaF_2_. As illustrated in Figure 4a,b, the reduction in the thickness of the first layer of VO_2_ results in an increase in both high-temperature emissivity and emissivity tunability. Additionally, when the second layer of VO_2_ is thinner, it more effectively enhances emissivity tunability. As shown in Figure 4c,d, the emissivity tunability initially increases and then decreases with the increase in BaF_2_ thickness, and the emissivity in both states exhibits an increasing trend. When the thicknesses of the first and second layers of VO_2_ are 10 nm and 20 nm, respectively, and the thickness of the first and second layers of BaF_2_ is 1400 nm and 1200 nm, the maximum tunability of emissivity is achieved, reaching 0.7767. In this structure, the high-temperature emissivity approaches 0.8945, representing a significant improvement compared to the single-period structure.

**Figure 5:**
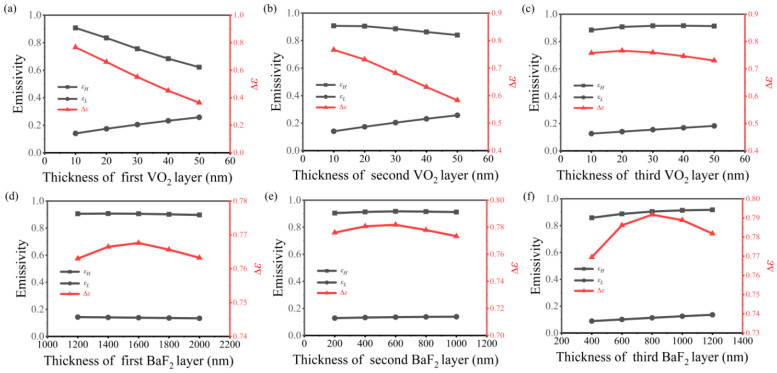
The IR emittance of high/low temperatures and the emissivity tunability of the VO_2_-based three-period structure varies with the thickness of the VO_2_ layer (**a**–**c**); the IR emittance of high/low temperatures and the emissivity tunability of the VO_2_-based three-period structure varies with the thickness of the BaF_2_ layer (**d**–**f**).

Figure 5 illustrates the emissivity tunability of a three-period structure based on the stacked FP multilayer film of VO_2_/BaF_2_. As observed in Figure 5a–c, the decrease in the thickness of VO_2_ in the first and second layers leads to an increase in both high-temperature emissivity and emissivity tunability. Additionally, when the third layer of VO_2_ is thinner, it effectively enhances emissivity tunability. As depicted in Figure 5d–f, the emissivity tunability initially increases and then decreases with the increase in BaF_2_ thickness, and the emissivity in both states shows an increasing trend. When the thicknesses of the first, second, and third layers of VO_2_ are 10 nm, 10 nm, and 20 nm, respectively, and the thicknesses of the first, third, and second layers of BaF_2_ are 1600 nm, 600 nm, and 800 nm, respectively, the maximum tunability of emissivity is achieved, reaching 0.7917. Compared with the dual periodic structure, the emissivity tunability is slightly improved. In addition, the four-period structure has also been optimized, with a maximum emissivity tunability of 0.7881, slightly lower than the three-period structure. The specific optimization process is shown in Figures S1 and S2. In summary, the three-period structure achieves the maximum emissivity tunability of the SRD.

### 3.3. Protection Layer

**Figure 6:**
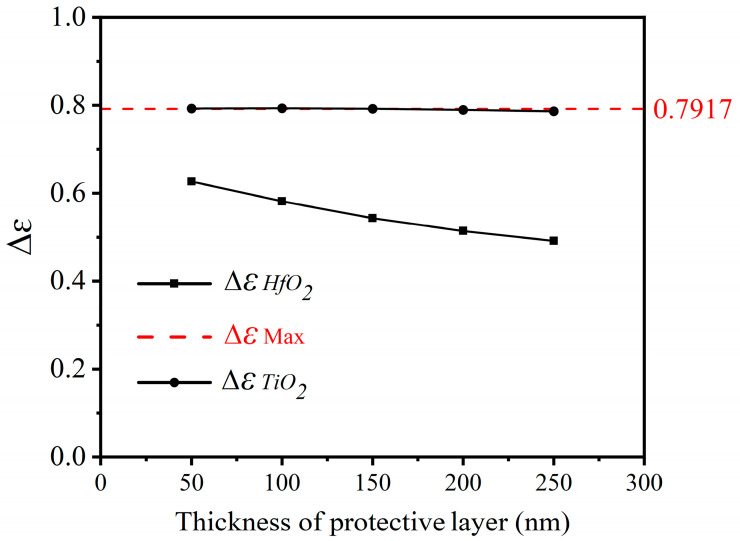
The emissivity tunability of the optimized three-period VO_2_-based structure with protective layers (HfO_2_ or TiO_2_), where the red dashed line indicates the maximum Δ*ε* without a protective layer.

In order to extend the operational lifespan of the device and protect the SRD structure from external oxidation, a protective layer is added to the three-period structure in this study. The influence of two infrared transparent films (HfO_2_ and TiO_2_) [33,34,35] on the emissivity tunability of the device is studied under the previously determined optimal thickness. As illustrated in Figure 6, it is clear that the HfO_2_ and TiO_2_ materials have distinct effects on the performance of the SRD. The addition of a HfO_2_ protective layer leads to a significant decrease in the tunability of the emissivity, with the reduction becoming more pronounced with the increase in layer thickness, thereby significantly impacting the SRD performance in comparison to the unprotected structure. In contrast, when TiO_2_ serves as the protective layer, the tunability of the emissivity shows slight fluctuations, with overall minimal change, indicating that TiO_2_ can effectively protect the SRD. It is worth noting that when the thickness of the TiO_2_ layer is 0.1 µm, the maximum emissivity tunability is achieved at 0.7932, demonstrating excellent emissivity tunability capability. Table 1 illustrates the performance comparison between our proposed structure and other relevant studies in the literature. As observed in Table 1, the structure designed in this work has the highest tunability of emissivity.

**Figure 7:**
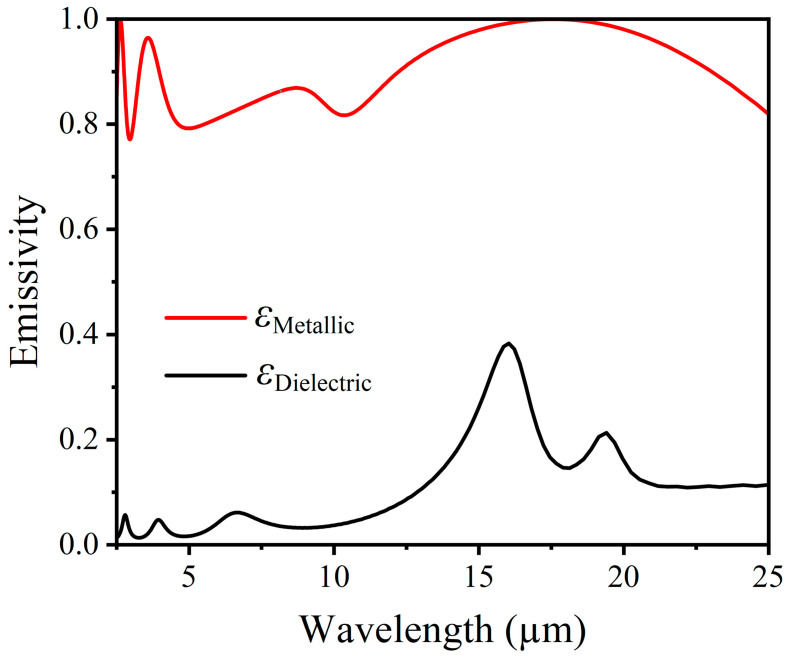
The IR emittance of a three-period structure with a protective layer varies with wavelength at both metallic and dielectric states.

In order to better understand the enhancement mechanism, Figure 7 illustrates the variation in infrared emissivity with a wavelength for the three-period structure with a protective layer at both high and low temperatures across the spectrum from 2.5 to 25 µm. It can be clearly seen from Figure 7 that in the metallic state of VO_2_, the emissivity of the stacked FP film is greater than 0.95 in the spectral range of 13 to 21 µm. Conversely, in the dielectric state of VO_2_, there is little to no emission or absorption in the range of 2.5 to 13 µm. Additionally, it can be observed that the dielectric state of VO_2_ exhibits peaks around 16.03 µm and 19.41 µm, which are attributed to the large imaginary part of the dielectric constant of VO_2_, leading to inherent absorption in the infrared emission spectrum [8,36]. Moreover, when VO_2_ is in the metallic state, peaks are present around 3.58 µm and 17.67 µm.

**Figure 8:**
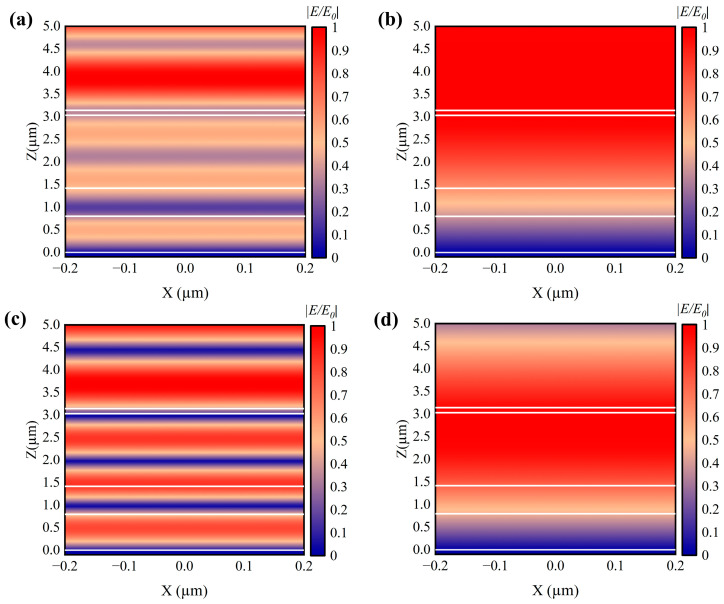
The normalized electric field |*E*/*E_0_*| is shown for metallic (**a**,**b**) and dielectric (**c**,**d**) states at wavelengths of 2.9527 and 17.67 µm. *E_0_* represents the incident electric field, directed perpendicular to each layer. The white lines represent the boundaries of each layer. In the figures, the air, TiO_2_, and BaF_2_ layers are clearly visible, while the VO_2_ layer is too thin to be represented.

Figure 8 illustrates the normalized electric field |*E*/*E_0_*| at wavelengths of 2.9527 and 17.67 µm for metallic (a,b) and dielectric (c,d) states. As shown in Figure 8a,c, at a wavelength of 2.9527 µm, the VO_2_ layer exhibits intense interference between incident and reflected waves in both states, resulting in a significantly enhanced and confined electric field compared to the incident electric field [37]. This interference is a result of the superposition of Fabry–Perot resonances within the stacked VO_2_/BaF_2_ layers. The first and third VO_2_ layers are located in a region where the electric field is almost zero, leading to a lower emissivity for the entire structure, as indicated by the valley at 2.9527 µm in Figure 7. Additionally, it is noticeable that the electric field distribution at high temperatures is similar to that at low-temperature states, but the enhanced electric field in the air domain is significantly weakened, primarily due to the reduced intensity of the reflected waves. For the metallic state at the resonant wavelength, as shown in Figure 8b, the perfect absorption caused by the superimposed FP resonance leads to a monotonically decreasing electric field along the Z-axis, with almost no interference between the incident and reflected waves [38]. This is consistent with the high emissivity observed in the metallic state, where the VO_2_ layer acts as a perfect absorber. When the VO_2_ layer is in the dielectric state, as depicted in Figure 8d, the entire VO_2_ and BaF_2_ layers become semi-transparent, and clear interference between the reflected and incident waves can be observed. The combination of these effects, particularly the superimposed Fabry–Perot resonances and the interference between the reflected and incident waves, is the key to achieving the high emissivity tunability of the proposed structure. This analysis provides a deeper understanding of the physical mechanisms and how they can improve device performance through smart thermal control.

## 4. Conclusions

In summary, we systematically studied the effects of dielectric materials (Al_2_O_3_, ZnS, and BaF_2_), layer thicknesses, and cyclic structures on the emissivity tunability of an SRD and proposed a VO_2_-based stacked FP multilayer film with protective layers. This structure is constructed by stacking three VO_2_/BaF_2_ layers on the Al substrate to form multiple FP resonances, achieving a maximum emissivity tunability of 0.7917, which represents a great enhancement over single-cycle sandwich structures. Furthermore, the protective layers (HfO_2_ or TiO_2_) were investigated to extend the operational lifespan of SRD under space conditions. The results showed that TiO_2_ with a thickness of 0.1 µm maintained the emissivity tunability at 0.7932, slightly higher than the unprotected structure. In order to better understand the enhancement mechanism, electric field analysis revealed that the mechanism of SRD was the result of the combined action of FP resonance and multiple solar reflections. The structure proposed in this paper exhibits higher emissivity tunability and a longer operational lifespan. Overall, this study is of great significance for the development of smart thermal control devices for spacecraft and provides important theoretical guidance for the design of thermal control systems. However, it is important to recognize the limitations of this approach. Stacking multilayer films increases a device’s complexity. And the fabrication of the multilayer films, particularly the precise control of the thickness of each film layer, presents technical challenges. Despite these challenges, the notable advantages of the proposed structure make it a promising model for further research and development.

## Figures and Tables

**Table 1:** Performance comparison with other previous relevant studies.

Reference	Materials and Structure	*ε_H_*	Δ*ε*
Hendaoui et al. [18]	VO_2_/HfO_2_/Ag	0.68	0.55
Wu et al. [22]	VO_2_/BaF_2_/Ag	0.78	0.64
Shrewsbury et al. [20]	VO_2_/ZnSe/VO_2_/ZnSe/Au	0.77	0.69
Zhang et al. [8]	Si/VO_2_/BaF_2_/Ag	0.65	0.75
This work	TiO_2_/VO_2_/BaF_2_/VO_2_/BaF_2_/VO_2_/Al	0.90	0.79

## Data Availability

Data are contained within the article and Supplementary Materials.

## Supplementary Materials

The following supporting information can be downloaded at https://www.mdpi.com/article/10.3390/nano14161348/s1, Figure S1: The IR emittance of high and low temperatures and emissivity tunability of the VO_2_-based four-period structure varies with VO_2_ layers; Figure S2: The IR emittance of high and low temperatures and emissivity tunability of the VO_2_-based four-period structure varies with BaF_2_ layers.
