# Supplementary material for: VO2-Based Spacecraft Smart Radiator with High Emissivity Tunability and Protective Layer
Source: Nanomaterials (Basel). 2024 Aug 15;14(16):1348. doi: 10.3390/nano14161348 (PMC11357278; doi:10.3390/nano14161348)
Supplement: Supplementary file 1 [file nanomaterials-14-01348-s001.zip › nanomaterials-3143038-supplementary.pdf]

Supporting information

VO_2-based spacecraft smart radiator with high emissivity tunability and protective layer

Qingjie Xu, Haining Ji*, Yang Ren, Yong Ouyang, Bin Liu, Yi Wang, Yongxing Chen, Peng Long, Cong Deng, Jingting Wang

School of Physics and Optoelectronics, Xiangtan University, Xiangtan, Hunan 411105, P. R. China

sdytjhn@126.com

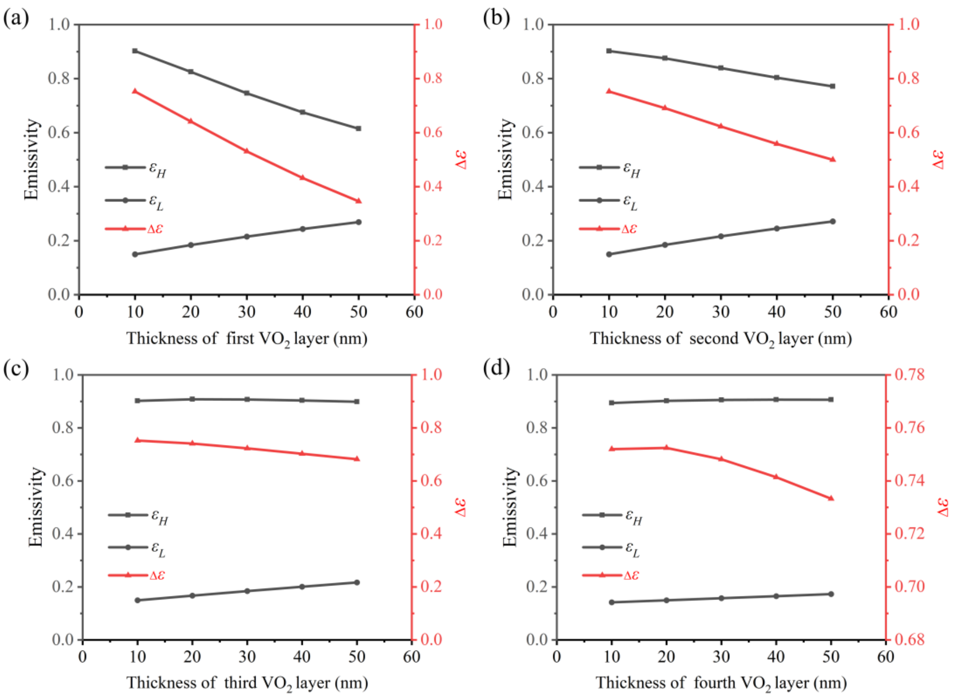

Figure S1. The IR emittance of high and low temperatures and emissivity tunability of the VO_2-based four-period structure varies with VO_2 layers.

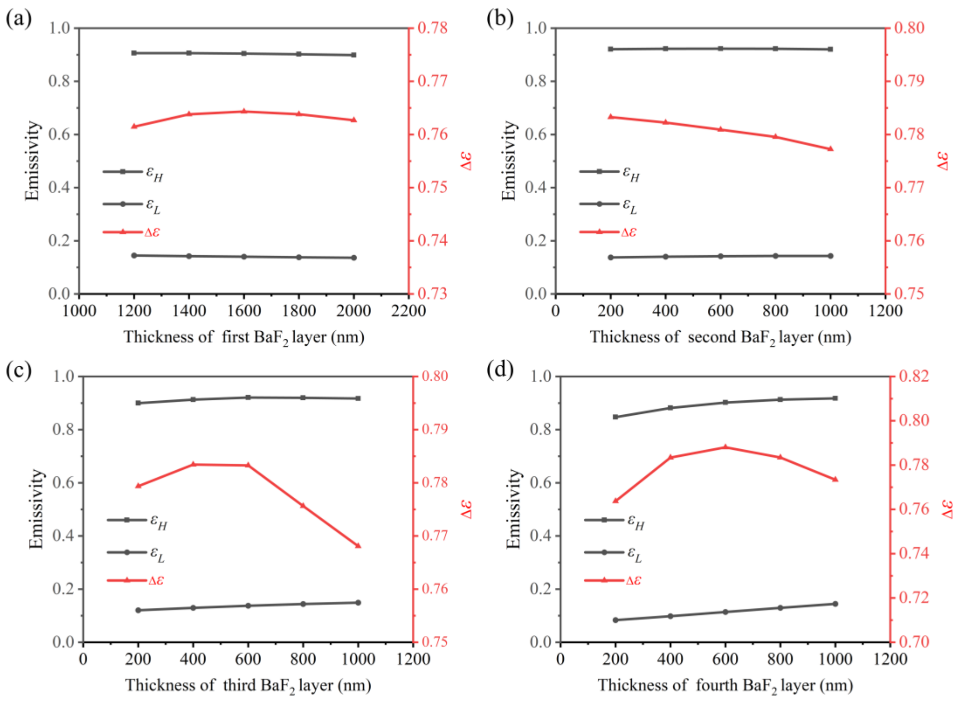

Figure S2. The IR emittance of high and low temperatures and emissivity tunability of the VO_2-based four-period structure varies with BaF_2 layers.
